# Stellate Ganglion Block for Treatment-Resistant Post-traumatic Stress Disorder: A Case Report

**DOI:** 10.7759/cureus.117912

**Published:** 2026-10-14

**Authors:** Elizabeth Francis

**Affiliations:** 1 School of Nursing, Duke University, Durham, USA

**Keywords:** c-ptsd, psychiatry and mental health, psycho pharmacology, ptsd (post-traumatic stress disorder), stellate ganglion

## Abstract

Post-traumatic stress disorder (PTSD) is a psychiatric condition that can develop following exposure to traumatic events and is characterized by intrusive memories, avoidance, negative changes in mood and cognition, and heightened arousal. A woman in her late 20s with bipolar I disorder, PTSD, and anorexia nervosa, binge-purge subtype, demonstrated minimal response to multiple medication trials targeting PTSD symptoms. Following administration of a stellate ganglion block (SGB), symptom scores decreased on the Clinician-Administered PTSD Scale for DSM-5 (CAPS-5) and the PTSD Checklist for DSM-5 (PCL-5), although residual symptoms remained. This case highlights the potential role of SGB as an off-label therapeutic option for PTSD.

## Introduction

Post-traumatic stress disorder (PTSD) is a psychiatric condition that may develop following exposure to actual or threatened death, serious injury, or sexual violence [1]. According to the Diagnostic and Statistical Manual of Mental Disorders, Fifth Edition, Text Revision (DSM-5-TR), PTSD involves symptoms across four major diagnostic clusters: intrusion, avoidance, negative alterations in cognition and mood, and alterations in arousal and reactivity [1,2]. Symptoms may include intrusive memories, nightmares, flashbacks, avoidance of trauma-related reminders, persistent negative emotional states, diminished interest in activities, hypervigilance, an exaggerated startle response, sleep disturbance, and difficulty concentrating [1]. Symptoms must persist for more than one month and cause clinically significant distress or functional impairment [1,2]. The presentation and severity of PTSD vary, and co-occurring psychiatric conditions can complicate assessment and treatment.

Evidence-based treatments for PTSD include trauma-focused psychotherapies, such as prolonged exposure (PE), cognitive processing therapy (CPT), and eye movement desensitization and reprocessing (EMDR), as well as selected pharmacologic interventions [1,2]. Despite these options, some patients continue to experience substantial symptoms following extensive treatment. Treatment planning can be particularly complex when PTSD co-occurs with other psychiatric conditions, including bipolar disorder and eating disorders, because clinicians must consider overlapping symptoms, medication tolerability, mood stability, and the effects of concurrent treatment.

Stellate ganglion block (SGB) is an interventional procedure involving the injection of a local anesthetic near the stellate ganglion, a sympathetic nerve structure in the cervical region [2,3]. SGB has been investigated as a potential adjunctive intervention for PTSD based on the hypothesis that modulation of sympathetic activity may influence hyperarousal and other trauma-related symptoms [2-5]. Although clinical studies have reported symptom improvement in some patients, randomized trials have produced mixed findings, and the procedure's role in PTSD treatment remains under investigation [3-6].

This case report describes changes in PTSD, depression, and anxiety symptom scores following SGB in a patient with longstanding PTSD, bipolar I disorder, and anorexia nervosa, binge-eating/purging type. The case is clinically relevant because the patient had received extensive psychiatric treatment, continued psychotherapy and medication management, and experienced substantial psychiatric comorbidity. The report also highlights the interpretive challenges involved in evaluating an adjunctive intervention when symptoms may be influenced by several concurrent and longitudinal factors.

## Case presentation

A woman in her late 20s with diagnoses of PTSD, bipolar I disorder, and anorexia nervosa, binge-eating/purging type, underwent SGB at an external pain management clinic. Her PTSD symptoms began approximately 15 years before the study, whereas symptoms associated with bipolar I disorder and anorexia nervosa began approximately 10 years before the study. PTSD was identified as her most distressing psychiatric concern and the primary target of the intervention.

The patient had received psychiatric treatment over several years across multiple levels of care, including inpatient psychiatric hospitalization, partial hospitalization, intensive outpatient treatment, outpatient psychiatric management, and psychotherapy. Treatment over time addressed her co-occurring psychiatric diagnoses rather than PTSD in isolation. Psychotherapeutic modalities received during her treatment history included cognitive behavioral therapy (CBT), dialectical behavior therapy (DBT), EMDR, PE, and CPT. At the time of the study, she continued regular psychiatric medication management and psychotherapy for all three diagnoses.

Her PTSD symptom presentation included intrusive memories and flashbacks, hyperarousal, sleep disturbance, impaired concentration and memory, and social withdrawal, with associated functional impairment. Her psychiatric history was also notable for bipolar I disorder and anorexia nervosa, binge-eating/purging type, which added complexity to treatment planning and interpretation of changes in mood, anxiety, and trauma-related symptoms.

Several months before the study, the patient experienced a mixed episode associated with bipolar I disorder, prompting an increase in risperidone. She had not experienced mania for five months before the study. At the start of the study, her medication regimen included risperidone 2 mg daily, quetiapine 25mg daily, clonidine 0.1 mg twice daily, lithium extended-release 600 mg daily, and lamotrigine 150 mg daily. The risperidone dose reflected the regimen in place during the study following the earlier increase. No additional psychiatric medication changes were made during the seven-week follow-up period.

Prior medication trials included prazosin, olanzapine, sertraline, Depakote, aripiprazole, venlafaxine, and fluoxetine. Previous selective serotonin reuptake inhibitor trials were limited by adverse effects and/or activating effects in the context of bipolar I disorder. The available clinical history did not establish the specific effect of each medication or indicate that every prior medication trial constituted an adequate treatment trial for PTSD.

Laboratory testing performed approximately one month before the study included a complete blood count (CBC), comprehensive metabolic panel (CMP), thyroid panel, iron and ferritin panel, vitamin B12, and folate. All results were within normal limits. These findings provided relevant medical context but did not exclude every possible medical or nutritional contributor to psychiatric symptoms. No significant medical history apart from the patient's psychiatric conditions was documented. 

No psychiatric medication changes occurred during the seven-week follow-up period. The patient continued psychotherapy and regular psychiatric medication management for her co-occurring diagnoses. No adverse effects were documented during psychiatric follow-up. Because the patient continued to receive psychiatric care and psychotherapy, and because the available observations were limited to a single case, the symptom changes cannot be attributed exclusively to SGB.

SGB was performed at an external pain management clinic as an adjunctive intervention targeting the patient's persistent PTSD symptoms. Following the procedure, the patient continued regular psychotherapy and psychiatric medication management. Her medication regimen remained unchanged throughout the seven-week follow-up period. We obtained written informed consent for publication of this case report.

PTSD, depressive, and anxiety symptoms were assessed at baseline and at seven-week follow-up using standardized measures. PTSD severity was assessed using the Clinician-Administered PTSD Scale for DSM-5 (CAPS-5) and the PTSD Checklist for DSM-5 (PCL-5) [7,8]. Depressive symptoms were measured using the Patient Health Questionnaire-9 (PHQ-9), and anxiety symptoms were assessed using the Generalized Anxiety Disorder-7 (GAD-7) [9,10]. 

At baseline, the patient's CAPS-5 score was 61, and her PCL-5 score was 67, indicating substantial PTSD symptom burden. Her baseline PHQ-9 score was 23, reflecting severe depressive symptoms, and her GAD-7 score was 20, reflecting severe anxiety symptoms.

At week seven, the CAPS-5 score decreased from 61 to 35, and the PCL-5 score decreased from 67 to 30. The PHQ-9 score decreased from 23 to 15, while the GAD-7 score decreased from 20 to 15. Symptom scores at baseline and seven-week follow-up are summarized in Figure 1. These changes represented reductions in measured PTSD, depressive, and anxiety symptoms. However, the follow-up scores indicated that clinically relevant symptoms remained.

**Figure 1:**
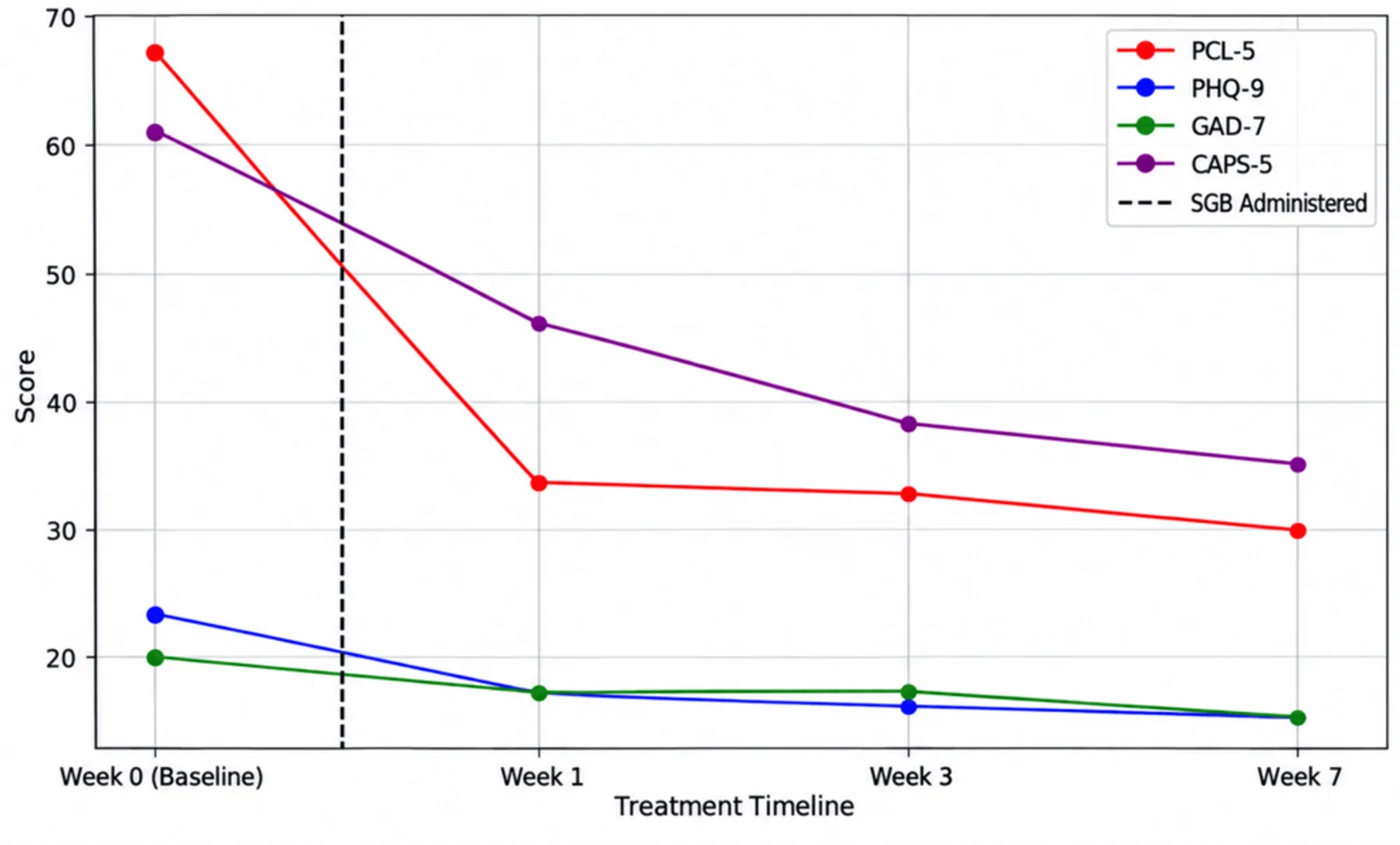
Symptom score improvement following SGB. PTSD, post-traumatic stress disorder; PCL-5, PTSD Checklist for DSM-5; PHQ-9, Patient Health Questionnaire-9; GAD-7, Generalized Anxiety Disorder-7; CAPS-5, Clinician-Administered PTSD Scale for DSM-5; SGB, stellate ganglion block

## Discussion

This case describes reductions in measured PTSD, depressive, and anxiety symptoms during the seven weeks following SGB in a woman with longstanding PTSD, bipolar I disorder, and anorexia nervosa, binge-purge subtype. The findings should be interpreted in the context of emerging but mixed evidence regarding SGB for PTSD. In a multisite randomized clinical trial, it was reported that participants who received two SGB procedures had significantly greater improvement in CAPS-5 scores than those who received sham treatment over eight weeks [3]. In contrast, another study found no statistically or clinically meaningful difference between SGB and sham treatment on PTSD outcomes in a randomized, double-blind trial [11]. A systematic review similarly identified promising findings from case reports and some clinical trials but emphasized the inconsistent results of randomized controlled trials and the need for additional research [4]. The symptom reductions observed in this case are consistent with literature suggesting that SGB may reduce PTSD symptom severity. One comprehensive review reported promising outcomes across military and civilian populations, particularly for hyperarousal symptoms [12]. In this patient, the PCL-5 score decreased from 67 to 30, and the CAPS-5 score from 61 to 35 by week seven, demonstrating improvement across two validated measures of PTSD severity. The reduction in symptom scores observed in the present case is consistent with improvement reported in some SGB studies; however, a single case cannot establish treatment efficacy or demonstrate that the observed changes were attributable to the procedure.

The case illustrates the complexity of treating PTSD in the context of multiple psychiatric diagnoses. PTSD symptoms preceded the onset of symptoms associated with bipolar I disorder and anorexia nervosa by approximately five years. This chronology is relevant to understanding the patient's longitudinal clinical presentation, although it does not establish that PTSD caused or contributed directly to the development of the other conditions. The coexistence of these diagnoses can complicate clinical assessment because sleep disturbance, concentration difficulties, anxiety, and mood changes may arise from more than one condition or be influenced by concurrent treatments.

The patient had engaged with several levels of psychiatric care, including inpatient hospitalization, partial hospitalization, intensive outpatient treatment, outpatient medication management, and psychotherapy. Her treatment history included CBT, DBT, EMDR, PE, and CPT. However, the available information did not establish the duration, sequence, adequacy, or individual effectiveness of each psychotherapy modality. Therefore, the case should not be interpreted as demonstrating that all established PTSD treatments had failed. Rather, it illustrates the clinical context in which an adjunctive intervention was pursued for persistent symptoms that the patient identified as her most distressing concern.

The patient's psychiatric comorbidities were considered when interpreting changes in PTSD symptom severity. Although bipolar I disorder can contribute to mood, sleep, and concentration symptoms, the patient did not meet criteria for a current manic or mixed episode at the time of the study. She had not experienced mania during the five months preceding SGB, although a mixed episode several months earlier had prompted an increase in risperidone. Her medication regimen remained unchanged throughout the seven-week follow-up. These findings reduce concern that an active manic or mixed episode accounted for the observed changes; however, residual mood symptoms and the longitudinal course of bipolar disorder could not be definitively excluded as contributing factors. Similarly, the patient's history of anorexia nervosa, binge-purge subtype, was considered when evaluating potential contributors to her psychiatric presentation.

Laboratory testing performed one month before the study, including CBC, CMP, thyroid function, iron and ferritin, vitamin B12, and folate, was within normal limits. These findings reduce concern for abnormalities in the specific laboratory parameters assessed at that time but do not exclude all possible medical, nutritional, or other contributors to psychiatric symptoms. The results should be interpreted as contextual clinical information rather than evidence that nonpsychiatric factors were definitively excluded. Accordingly, the observed changes in PTSD symptom scores should be interpreted in the context of these comorbidities rather than attributed exclusively to SGB.

The concurrent reductions in depression and anxiety scores are also noteworthy. They raise questions about whether symptom changes following SGB may extend beyond PTSD-specific measures or reflect broader changes in psychological distress. However, the PHQ-9 and GAD-7 findings cannot establish that SGB directly improved depressive or anxiety disorders. Changes in trauma-related distress, ongoing psychotherapy, the course of co-occurring conditions, and other clinical factors may have contributed to the observed results. Future studies should assess these outcomes separately rather than assuming that improvements across symptom measures represent a single treatment effect.

Clinically, this case reinforces the importance of maintaining comprehensive psychiatric care when considering interventional approaches to PTSD. SGB was used as an adjunct to, rather than a replacement for, ongoing psychotherapy and medication management. The absence of documented adverse effects during psychiatric follow-up is reassuring within the limits of this report, but it does not establish the procedure's safety more broadly. SGB is an invasive procedure with potential complications, including bleeding, infection, nerve injury, and unintended local anesthetic effects [3-5]. Decisions about its use require consideration of the available evidence, procedural risks, patient preferences, and the continued role of established PTSD treatments.

This report should be viewed as hypothesis-generating. It does not establish efficacy, identify which patients are most likely to respond, or support recommending SGB as a standard treatment for PTSD. Nevertheless, detailed case observations may help inform future investigations of SGB in patients with complex psychiatric presentations, particularly when longitudinal clinical information and concurrent treatments are clearly documented.

Future research should evaluate SGB in larger and more diverse populations, including patients with complex psychiatric comorbidities who may be underrepresented in clinical trials. Randomized controlled trials comparing SGB with sham procedures or appropriate control conditions would help clarify the extent to which symptom changes are attributable to the procedure rather than expectancy effects, nonspecific treatment effects, or other influences. Longer follow-up is also needed to determine whether improvements persist beyond the initial weeks after treatment, whether symptoms fluctuate or recur, and whether repeat procedures are associated with additional benefit or risk. Standardized reporting of procedural techniques, including the approach used and the anesthetic agent and dose, would improve reproducibility and allow comparisons across studies. Systematic documentation of adverse events is essential to evaluate the safety profile of SGB and support informed clinical decision-making.

Research should also examine potential predictors of response. Relevant variables may include baseline PTSD severity, duration of illness, previous treatment exposure, symptom profile, and co-occurring psychiatric diagnoses. Studies specifically enrolling patients with bipolar disorder and eating disorders could help determine whether findings from broader PTSD populations generalize to patients with these conditions. In populations with bipolar disorder, monitoring should include mood symptoms and potential mood destabilization, with careful documentation of medication regimens, medication changes, and the course of the underlying illness.

Finally, comparative-effectiveness research may help establish how SGB fits within existing PTSD treatment pathways. Studies could evaluate whether SGB offers additional benefit when added to established trauma-focused psychotherapy or pharmacologic treatment, and identify circumstances in which the potential benefits may justify procedural risks and costs. Until higher-quality evidence clarifies its effectiveness, durability, and appropriate patient selection, SGB should not be considered a substitute for established PTSD care. This case provides a clinically detailed observation to inform these research questions without establishing a causal treatment effect.

Several limitations must be considered when interpreting this case. First, this is a single-patient report, and the findings cannot be generalized to the broader PTSD population. Without a control condition, it is not possible to determine whether the observed symptom reductions resulted from SGB, nonspecific treatment effects, expectancy, natural symptom fluctuation, or other factors. The patient continued psychotherapy and regular psychiatric medication management throughout the follow-up period. Although no psychiatric medication changes occurred during the seven weeks after SGB, the patient had experienced a mixed episode several months before the study that prompted an increase in risperidone. The influence of that earlier adjustment and the natural course of her psychiatric conditions cannot be excluded. The patient's psychiatric comorbidity complicates interpretation of changes in depressive and anxiety symptoms. The PHQ-9 and GAD-7 scores improved alongside PTSD scores, but these measures do not establish the cause of those changes or whether they were directly related to improvement in PTSD symptoms. The patient's history of bipolar I disorder and anorexia nervosa, binge-eating/purging type, further underscores the importance of considering overlapping symptoms and the longitudinal course of each condition. Although the patient had received several psychotherapeutic modalities and multiple levels of care, the available information did not establish the duration, sequence, fidelity, or specific effectiveness of each intervention. Consequently, the report cannot establish that every evidence-based PTSD treatment had been adequately attempted or had failed. Finally, the seven-week follow-up provides only a short-term view of the patient's clinical course. Longer observation would be needed to evaluate the durability of symptom changes, recurrence of symptoms, and any need for additional intervention.

## Conclusions

This case describes reductions in PTSD, depressive, and anxiety symptom scores during the seven weeks following SGB in a woman in her late 20s with longstanding PTSD, bipolar I disorder, and anorexia nervosa, binge-eating/purging type. PTSD was her most distressing psychiatric concern and the primary target of the procedure. She had a substantial history of psychiatric treatment and continued psychotherapy and medication management throughout follow-up. This case suggests that SGB may offer meaningful benefit as an adjunctive, off-label intervention for treatment-resistant PTSD, even in the context of significant psychiatric comorbidity. The case highlights the potential value of studying SGB as an adjunctive intervention in patients with complex psychiatric presentations, while underscoring the need for larger controlled trials, longer follow-up, standardized procedural reporting, and specific evaluation of patients with psychiatric comorbidities. Until stronger evidence is available, SGB should be considered investigational in this context and should not replace comprehensive, evidence-based PTSD treatment.

These results are consistent with emerging evidence that SGB can produce rapid, well-tolerated symptom relief in patients with treatment-refractory PTSD and prominent autonomic hyperarousal, via a mechanism distinct from standard pharmacotherapy. However, as a single case report without long-term follow-up or the ability to isolate SGB's effect from concurrent psychotherapy, these findings are hypothesis-generating rather than conclusive. Larger controlled trials are needed to define optimal patient selection, treatment protocols, and durability of response before SGB can be considered a validated option for treatment-resistant PTSD.
